# Mr. Brunel

**Published:** 1859-11

**Authors:** 


DEATH OF MR. BRUNEL.—The death of this illustrious engineer, which recently occurred at London, at the age of fifty-three, has recalled to our recollection the great suffering which he endured, a number of years ago, in consequence of the inhalation of a half sovereign while engaged in amusing some children in a trick at legerdemain. He was a native of Portsmouth, England, and was a man of great industry and genius, as is proved by the fact that his name is associated with some of the most wonderful performances in machinery and architecture of the present century. The following is an abstract of Mr. Brunel's case, as recorded in the senior Editor's Treatise on Foreign Bodies in the Air-Passages, published in 1854:—

"On the 3d of April, 1843, Mr. Brunel, of London, in amusing some children, immediately after dinner, placed a half sovereign in his mouth, which by some accident slipped behind the tongue, causing a violent fit of coughing and choking, and the forcible ejection of the contents of the stomach. He strained two or three times afterwards, but did not again vomit. In the course of the evening he coughed at intervals, though not violently; and for the next twenty-four hours he complained of some stiffness and soreness in the throat. During the next two days, he experienced little or no inconvenience; he had no cough, and he employed himself as usual. On the 6th of April the cough returned, and on the 9th it became aggravated, apparently from exposure to cold. He expectorated some mucus slightly tinged with blood, and experienced a pain in the side of the chest, in the situation of the lower portion of the right bronchial tube. An aperient, followed by vomiting, greatly relieved these symptoms; but on the 11th of April, the cough was again troublesome, and on the 17th it was much increased by exposure to a cold, easterly wind. On the 19th, the patient was placed in the prone position, with his sternum resting on a chair, and his head and neck inclining downward, when he immediately had a distinct perception of a loose body slipping forward along the trachea. A violent convulsive cough ensued. On resuming the erect posture, he again experienced the sensation of a loose body moving in the trachea, but in the opposite direction—that is, toward the chest On the 25th, a somewhat similar experiment was made. At first, no cough ensued; but on the back, opposite the right bronchial tube, having been struck with the hand, the patient began to cough violently. The half sovereign, however, did not make its appearance. This process was twice repeated with no better result; and, on the last occasion, the cough was so distressing, and the appearance of choking so alarming, that it was evident that it would be imprudent to proceed further with this experiment, unless some precaution were used to render it more safe.

"On the 27th of April, tracheotomy was performed, and an attempt made, but without success, to reach the coin with the forceps introduced through the artificial opening. The contact of the instrument always induced the most violent convulsive coughing. The coin could not even be felt. On the 2d of May, these trials were repeated with the same result. Determined to desist from all attempts of this kind, the patient was permitted to rest until the 13th of May, when, being placed in the prone position, upon a platform made movable on a hinge in the center, the shoulders and body were fixed by means of a broad strap, and the head was lowered until the platform was brought to an angle of about 80 degrees with the horizon. The back was now struck with the hand; two or three efforts to cough followed, and presently he felt the coin quit the bronchial tube, striking almost immediately afterwards against the incisor teeth of the upper jaw, and then dropping out of the mouth. A small quantity of blood, drawn into the trachea from the granulations of the external wound, was at the same time ejected; no spasm in the muscles of the glottis took place, and there was none of the inconvenience and distress which had caused so much alarm on the former occasion. A speedy recovery was the consequence. It is worthy of remark that the stethoscope, which was repeatedly applied to the chest during the progress of this case, detected no difference in the state of the respiration."

